# The medial frontal-prefrontal network for altered awareness and control of action in corticobasal syndrome

**DOI:** 10.1093/brain/awt302

**Published:** 2013-11-29

**Authors:** Noham Wolpe, James W. Moore, Charlotte L. Rae, Timothy Rittman, Ellemarije Altena, Patrick Haggard, James B. Rowe

**Affiliations:** 1 Department of Clinical Neurosciences, University of Cambridge, Cambridge CB2 0SZ, UK; 2 Medical Research Council Cognition and Brain Sciences Unit, Cambridge CB2 7EF, UK; 3 Department of Psychology, Goldsmiths, University of London, London SE14 6NW, UK; 4 Institute of Cognitive Neuroscience, University College London, London WC1N 3AR, UK; 5 Behavioural and Clinical Neuroscience Institute, University of Cambridge, Cambridge CB2 3EB, UK

**Keywords:** corticobasal syndrome, alien limb, apraxia, voluntary action, volition, pre-supplementary motor area

## Abstract

The volitional impairments of alien limb and apraxia are a defining feature of the corticobasal syndrome, but a limited understanding of their neurocognitive aetiology has hampered progress towards effective treatments. Here we combined several key methods to investigate the mechanism of impairments in voluntary action in corticobasal syndrome. We used a quantitative measure of awareness of action that is based on well-defined processes of motor control; structural and functional anatomical information; and evaluation against the clinical volitional disorders of corticobasal syndrome. In patients and healthy adults we measured ‘intentional binding’, the perceived temporal attraction between voluntary actions and their sensory effects. Patients showed increased binding of the perceived time of actions towards their effects. This increase correlated with the severity of alien limb and apraxia, which we suggest share a core deficit in motor control processes, through reduced precision in voluntary action signals. Structural neuroimaging analyses showed the behavioural variability in patients was related to changes in grey matter volume in pre-supplementary motor area, and changes in its underlying white matter tracts to prefrontal cortex. Moreover, changes in functional connectivity at rest between the pre-supplementary motor area and prefrontal cortex were proportional to changes in binding. These behavioural, structural and functional results converge to reveal the frontal network for altered awareness and control of voluntary action in corticobasal syndrome, and provide candidate markers to evaluate new therapies.

## Introduction

The ability to act voluntarily is fundamental to human life, yet it can be severely impaired by disease. An important example is the corticobasal syndrome (CBS), a complex movement disorder that often results from diffuse degeneration in cortical and subcortical areas (Gibb *et al.*, 1989; Rinne *et al.*, 1994). Clinical diagnostic criteria for CBS (Kumar *et al.*, 1998; Litvan *et al.*, 2003; Armstrong *et al.*, 2013) include two disorders of voluntary action: alien limb, the performance of semi-purposeful movements in the absence of volition; and apraxia, the inability to perform purposeful actions despite recognition of the goal of the action.

Alien limb and apraxia are archetypal disorders of volition, including abnormalities in voluntary motor control and in the conscious experience that normally accompanies voluntary action. However, little is known about the neural and behavioural mechanisms for these changes in control and awareness. Recent neuroimaging and lesion studies in healthy humans and non-human primates have revealed distinct frontoparietal networks for voluntary actions (Haggard, 2008), with particular interest in the pre-supplementary motor area (pre-SMA) in the medial frontal cortex (Passingham *et al.*, 2010).

The pre-SMA has been implicated in several aspects of voluntary action, particularly during the decision and preparation to act (Cunnington *et al.*, 2002). Moreover, the conscious experience that accompanies voluntary actions has been repeatedly linked to the pre-SMA. Neuronal activity in the pre-SMA is related to the awareness of intentions to act (Fried *et al.*, 2011), whereas direct electrical stimulation of this area can elicit an ‘urge’ to move a specific body part (Fried *et al.*, 1991). However, it remains unresolved how the pre-SMA mediates awareness of action.

The pre-SMA interacts with many cortical and subcortical areas (Nachev *et al.*, 2008), providing a range of possible networks as the neural substrate of awareness of action. The connections between the pre-SMA and dorsolateral prefrontal cortex are of particular importance, as functional connectivity between these areas increases with conscious intentions (Lau *et al.*, 2004). Furthermore, pre-SMA projections to basal ganglia can influence behaviour according to desired goals (Alexander and Crutcher, 1990), and have been proposed to support the conscious experience of ‘will’ (Haggard, 2008). The insular cortex has also been associated with the sense of control of actions in many neuroimaging studies (Farrer and Frith, 2002), and its interactions with the pre-SMA may also be crucial for the experience of volition (Hallett, 2007). Lastly, through its connections with premotor areas (Luppino *et al.*, 1993), the pre-SMA may influence a premotor-posterior parietal network that has been causally linked to the experience of conscious intentions to act (Desmurget *et al.*, 2009).

How might a neural network that is centred on the pre-SMA contribute to awareness of action, and its disorder in CBS? In an extension of motor control theory, the awareness and control of action are tightly linked: both are provided by internal models in the CNS (Frith *et al.*, 2000; Blakemore *et al.*, 2002). Initially, volitional signals drive the generation of motor commands according to the current goal. An ‘efference copy’ of motor commands allows a forward model to predict the sensory effect of a movement, which is compared with the actual sensory feedback. If the feedback matches the prediction, the sensory event is experienced as self-caused, but otherwise it is perceived as externally generated, independent of one’s own volition. The pre-SMA has been linked to this process, by providing the control signals that drive voluntary action, and/or efference copy signals and sensory predictions (Eagleman, 2004). Changes in a cortical network centred on the pre-SMA should thus cause disorders in both awareness and control of voluntary action by degrading the internal signals that drive voluntary actions, or by adding noise to the prediction signals.

Several studies have investigated the mechanisms of abnormal voluntary action in CBS, emphasizing insights from behavioural experiments and case studies (Riddoch *et al.*, 1998; McBride *et al.*, 2013). To understand better the effects of CBS on voluntary action, three key experimental components need to be jointly addressed: a quantitative and objective measure of awareness of action; a mechanistic account that draws on motor control theory; and systematic structural and functional neural information. The present study aims to combine these three methods.

The ‘intentional binding’ paradigm provides a quantitative experimental measure of the awareness of action or sense of ‘agency’ (Haggard *et al.*, 2002). The term agency in this context does not refer to a general belief that one is a distinct agent from others, but the specific subjective experience that one is responsible for making a particular action, and thereby its consequences. Intentional binding occurs when a voluntary action causes a sensory effect, such as a tone: voluntary actions are perceived to occur later than when actions are not followed by tones. Conversely, tones caused by voluntary actions are perceived to occur earlier than when they occur at random. Intentional binding can probe the experience of individual voluntary actions by measuring the perception of time of action and its effect (Moore and Haggard, 2008; Walsh and Haggard, 2013).

The perceived temporal attraction between actions and their sensory effects does not occur for involuntary (Haggard *et al.*, 2002) or passive movements (Engbert *et al.*, 2008), and is therefore a distinct feature of ‘volitional’ actions. As intentional binding does not rely on subjective accounts or introspection, it has been widely used in health and disease as an objective marker of awareness of voluntary action (Moore *et al.*, 2010*a*). Importantly, binding relies on both predictive and inferential processes that are contingent on distinct mechanisms in motor control. For example, binding draws upon internal volitional signals and a prediction mechanism for perceiving the result of one’s own action (Waszak *et al.*, 2012; Wolpe *et al.*, 2013), and therefore might be able to explain the nature of impaired motor control in patients (Voss *et al.*, 2010).

To identify the network changes for altered awareness and control of volitional action, we used complementary neuroimaging techniques: first localizing effects, and validating the pre-SMA involvement in volitional deficits in patients using structural grey and white matter analyses; then examining the changes in interactions of the pre-SMA using functional connectivity.

As intentional binding relies on distinct processes in motor control, the nature of binding abnormalities in CBS can give insights not only into awareness of action, but also into underlying deficits in voluntary motor control. We made three principal predictions. As CBS typically presents asymmetrically, we first predicted the experience -of- volitional actions in patients would be impaired specifically in the clinically more-affected hand, producing more abnormal binding than for the less-affected hand. Second, awareness of voluntary action as measured by binding, would correlate with changes in brain structure, including grey matter in the pre-SMA, and white matter tracts connected to the pre-SMA. Third, the role of the pre-SMA would be expressed as part of a broader cortical network, which may include reciprocal functional interactions with prefrontal, insula, parietal or premotor cortex.

## Materials and methods

### Participants

Ten patients (four female) aged 52–83 years [mean: 69 years, standard deviation (SD): 10] meeting clinical diagnostic criteria for CBS (Mathew *et al.*, 2012) were recruited from the specialist Disorders of Movement and Cognition clinic at the Cambridge University Hospitals NHS Trust. In addition, 16 (six female) age- and sex-matched controls aged 52–75 years (mean: 64 years, SD: 7) were included in the study and were compensated £30 plus travel expenses. The study was approved by the Cambridge Research Ethics Committee, and all participants signed a written informed consent before starting the experiments.

Cognitive abilities were assessed through the Mini-Mental State Examination in patients and control subjects (Folstein *et al.*, 1975) and Addenbrooke’s Cognitive Examination (Mioshi *et al.*, 2006). Patients with severe visual or communication deficits were excluded from the study, in view of the behavioural task. In patients, parkinsonian motor symptoms were assessed with the Unified Parkinson’s Disease Rating Scale-III motor subscale (Fahn and Elton, 1987). Alien limb phenomena were assessed by a 13-point structured questionnaire delivered verbally to the patient with caregiver or spouse corroboration. The questions were directed to the performance of both unwilled actions (‘anarchic hand phenomenon’) and impairments of authorship and sense of belonging of actions (Marchetti and Della Sala, 1998). These covered mirror movements, spontaneous face touching and levitation movements, perceived restlessness, unwanted reaching, oppositional reaching, inter-manual interference, restraint and the sense of ownership and control. The severity of limb apraxia was assessed through a structured examination by neurologists experienced in assessing patients with CBS (J.B.R. and T.R.), asking patients to copy three manual gestures and pantomime three transitive and three intransitive actions with their more-affected hand, as well as copy two bimanual gestures. Each successful gesture was scored 1 point (maximum score 11).

### Intentional binding procedure and analyses

**Figure 1:**
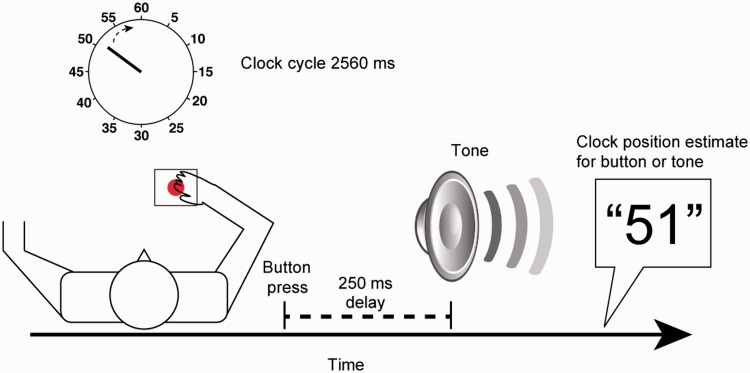
Illustration of the experimental behavioural procedure. Participants attended a clock and were asked to either press a button at their own pace or listen to a tone occurring at random, and then report the time of the event in -these- baseline conditions. The means of these baseline estimation errors were subtracted from those in the corresponding operant conditions, when the button press was followed by the tone. On any given trial, participants reported either the time of action or tone.

Participants performed the intentional binding task (Fig. 1; Haggard *et al.*, 2002). They attended a clock on a computer screen marked with numbers from 5 to 60 in intervals of five. A single hand rotated clockwise (period 2560 ms), providing a reference for the perceived time of events. Participants judged the time of self-paced button presses (4-cm button) or tones (400 Hz, 70-ms duration) and reported them verbally. They were discouraged from pre-planning the time at which they would press the button.

The clock hand started at a random position in each trial, and stopped at random 1500–2500 ms after the event that was judged. In the baseline conditions, times of actions (baseline action) and tones (baseline tone) were judged in isolation. In the operant conditions, tones followed the button presses by 250 ms. Participants were instructed in advance whether to judge the time of actions (operant action) or tones (operant tone). At the end of the operant conditions, participants were asked which of the two events they had in fact been judging, to confirm they were not confused about the task demands, despite the potential cognitive deficits in CBS. The four conditions were blocked in pseudo-randomized order, with the limitation that the study started and finished with baseline blocks. Each baseline block consisted of 10 trials, and each operant block had 20 trials (total of 20 trials per condition).

The mean time estimation shifts in operant conditions were separately compared against the corresponding baseline conditions to obtain action and tone binding measures, respectively. After completing the task with one hand, participants performed the task with the other hand. To minimize fatigue effects on the more-affected hand, patients were first tested with their more-affected hand. In controls, hand order was counterbalanced across participants. The behavioural session lasted 1 h.

We performed two complementary analyses, submitting action and tone binding measures to a mixed-design ANOVA. First, we examined the differences in binding between the two hands in patients and controls, with event (action versus tone) and hand (first versus second hand tested) as within-subject factor and group (patients versus controls) as between-subject factor. To examine the effects of age and Mini-Mental State Examination, these values were included as covariates in a subsidiary analysis. Lastly, action binding measures in the more-affected hand in patients were correlated with clinical measures of the affected hand: Unified Parkinson’s Disease Rating Scale, alien limb phenomena and apraxia, using Spearman’s ranked correlation.

### Structural magnetic resonance imaging acquisition and analysis

In a separate session, all controls and eight patients were scanned with a Siemens Tim Trio 3 T MR. T_1_-weighted MPRAGE images were acquired (repetition time = 2300 ms, echo time = 2.86 ms, field of view = 240 mm, flip angle = 9°, isotropic 1.25 mm voxels). Skull-stripping and brain extraction were performed with a pipeline optimized for neurodegenerative disease (Acosta-Cabronero *et al.*, 2008).

Subsequent preprocessing and analysis used Matlab 7 (Mathworks) with SPM8 (http://www.fil.ion.ucl.ac.uk/spm) for voxel-based morphometry (Ashburner and Friston, 2000), with Diffeomorphic Anatomical Registration Through Exponentiated Lie Algebra (DARTEL). The resulting study specific template was registered to Montreal Neurological Institute (MNI) space for normalization, followed by modulation and smoothing with an 8-mm full-width at half-maximum Gaussian kernel. Multiple regression analysis was performed to create a statistical parametric map of changes in local grey matter volume. Age and total intracranial volume were used as confounding covariates. Demeaned left hand action binding measures were covariates of interest. Left hand binding was used, as the laterality of disease in all but one patient was the left side. However, we note that even when using action binding from that individual patient’s more-affected right hand, the principal results remain.

Whole-brain group differences are reported at *P < *0.001 threshold with a minimum cluster size of 40 voxels, replicating the approach in previous volumetric studies in CBS (Josephs *et al.*, 2008). We also defined *a priori* a region of interest, encompassing the medial frontal and the medial prefrontal cortex (Passingham *et al.*, 2010), based on coordinates reported in (Amodio and Frith, 2006) for ‘action monitoring’, extending from the midline 8 mm laterally to each hemisphere. Correlations are reported at *P < *0.05, family-wise error (FWE) corrected for multiple comparisons. Significant voxels were localized according to the Oxford-Harvard cortical atlas in the FMRIB Software Library (FSL).

### Diffusion-weighted imaging acquisition and analysis

At the same scanning session, eight patients and 14 control subjects, were scanned with a diffusion-weighted imaging sequence (interleaved slices, repetition time = 7800 ms, echo time = 90 ms, field of view = 192 mm, isotropic 2 mm voxels, 63 gradient directions, b-value 1000 s/mm^2^). Data were preprocessed and analysed using FSL version 4.1.7 (www.fmrib.ox.ac.uk/fsl). Diffusion-weighted images were corrected for eddy currents and subject motion by affine registration to the b0 image, using the FSL ‘eddy_correct’ function. Diffusion tensors were linearly fitted using FSL ‘dtifit’, giving output maps of fractional anisotropy and mean diffusivity. Tract-based spatial statistics (http://www.fmrib.ox.ac.uk/fsl/tbss/) were used (Smith *et al.*, 2006). Individual fractional anisotropy images were registered to a common template (the most ‘representative’ subject; in this case a control subject) and co-registered to MNI space for display and report purposes. The mean fractional anisotropy skeleton was created at a threshold of fractional anisotropy >0.2. Subjects’ MNI-registered fractional anisotropy images were projected onto the skeleton. For analysis, the design matrix was similar to that used in the voxel-based morphometry analysis, except that age only was added as a covariate. Statistical testing was performed on each skeleton voxel, using non-parametric randomization tests (5000 permutations) with FSL tool ‘randomise’. Threshold-free cluster enhancement (Smith and Nichols, 2009) was applied for correction for multiple comparisons. Results reported are at *P < *0.05, FWE corrected, unless stated otherwise.

### Resting state functional imaging acquisition and analysis

Functional MRI images were obtained at the same scanning session using echo-planar imaging sensitive to the blood oxygen level-dependent signal (repetition time = 2000 ms, echo time = 30 ms, flip angle = 78°, field of view = 192°, 3 × 3 × 3.75 mm voxels). One hundred and fifty-five volumes were acquired (33 slices each), with eyes open in a dark bore with a blank screen. Two control subjects were excluded because of excess motion (gross movement >3 mm; >3° rotation). Preprocessing used a study-specific template from Visualization Toolkit software (http://www.vtk.org/) version 2.0.0. Subject’s MPRAGE scan was co-registered to MNI template, using affine transformation before sequentially transforming each subject's structural image to the group average, and combining these to construct a new average image. This step was repeated three times to create a closer approximation to the group average. Structural scans were normalized to the study specific template using FSL Non-Linear Image Registration Tool. Following slice timing and motion correction, each individual’s skull-stripped functional scan was co-registered to their structural scan and warped to the study specific template with a final resolution of 2 × 2 × 2 mm. Non-linear noise reduction using the FSL SUSAN tool (brightness threshold 500, spatial size 8 mm) and a high pass filter of 0.01 Hz were applied.

A voxel-wise seed-based connectivity analysis was performed using FSL ‘dual_regression’ function (Filippini *et al.*, 2009) as follows: significant pre-SMA and medial prefrontal voxels from the voxel-based morphometry results (where grey matter correlated with action binding in patients at *P < *0.001, uncorrected; total of 145 voxels) were used as a spatial map, and were warped into the study-specific template. The dual regression analysis first fit the data with a linear model using the spatial map as a spatial regressor to identify the associated temporal dynamics. To find a subject-specific map, the time courses were used as temporal regressors for an additional regression analysis. This resulted in pairs of matrices, which together model the spatial maps’ data. A single 4D data set of these estimates was created, and submitted to permutation testing, as in the analysis of the diffusion-weighted imaging data, resulting in spatial maps that show group differences and correlation with action binding measures in patients. Results reported are at *P < *0.05, FWE corrected. For display and report purposes, the results were registered back into MNI space.

The signal was not adjusted for non-neuronal physiological noise (respiratory and cardiac) during preprocessing or analysis, in part because of the time constraints and intrusiveness of physiological monitoring for this patient population. We note that CBS does not typically cause autonomic features that could alter respiratory or cardiovascular variability. Importantly, our analyses examine connectivity changes with a small region of interest in a between-group analysis, and identified a correlation between functional connectivity and binding within the patient group. These contrasts are less likely to be biased by physiological fluctuations.

## Results

### A specific abnormality in the perception of action in corticobasal syndrome

Patients with clinical diagnostic criteria of CBS (Table 1) and age-matched control were tested with the ‘intentional binding’ task. The mean perceived times of key presses and tones in the operant conditions were compared against those in the respective baseline conditions: presses made without eliciting tones, or tones occurring at random without preceding key presses (Fig. 1). The main analysis focused on how binding differed between hands in patients and controls (perceived times for all conditions summarized in Supplementary Table 1). The less-affected hand provided an important internal control for confound, such as visuospatial or attentional deficits in patients.

**Table 1:** Clinical details of patients with CBS participating in the study

Patient	Gender	Age, years	Disease duration, years	UPDRS-motor subscale	Alien limb score (0–13)	Apraxia score (0–11)	Cortical sensory loss	Motor features				Medication
								Akinesia	Dystonia	Myoclonus	Rigiditiy	
1	M	52	6	51	3	9	-	+	+	-	+	L, Ca
2	F	76	5	20	0	6	+	+	+	-	+	A
3	M	61	6	13	4	4	+	-	+	+	+	-
4	F	59	6	12	0	4	+	-	+	+	+	Clon
5	M	79	4	14	1	2	+	+	+	-	+	L, Ca
6	M	70	4	24	3	8	-	-	+	-	-	A
7	M	83	3	23	5	0	+	+	+	-	+	L, Ca
8	F	69	4	20	0	7	+	-	+	+	+	L, Ca
9	M	74	4	50	5	1	+	+	+	+	+	L, Ca
10	F	71	3	23	4	*	+	+	+	-	+	A, L, Ca, Clon

A = amantidine; Ca = carbidopa; Clon = clonazepam; L = levodopa; UPDRS = Unified Parkinson’s Disease Rating Scale.

*Apraxia could not be reliably scored because of severe dystonia.

**Figure 2:**
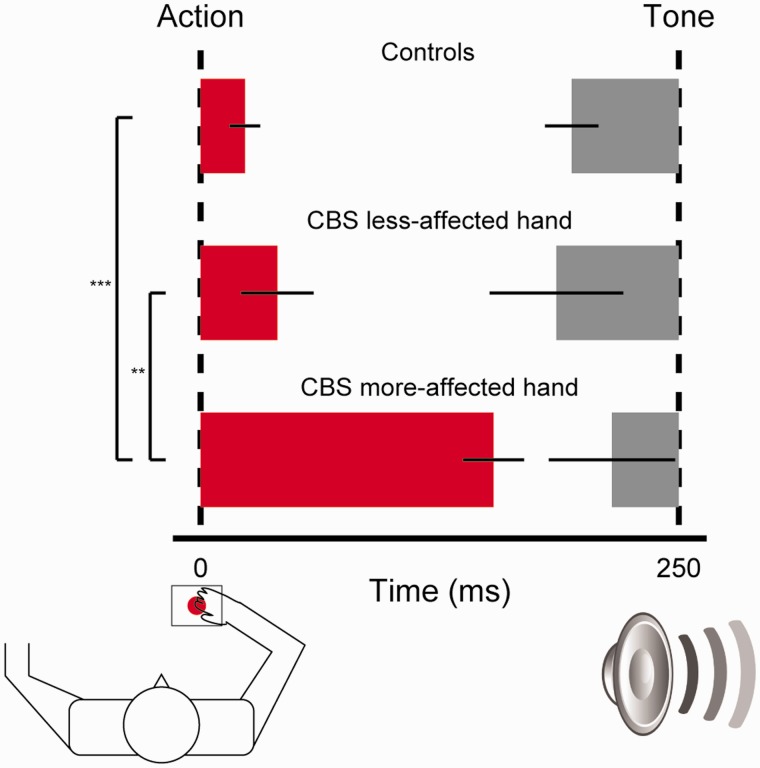
Action and tone binding in controls and patients with CBS. The bar chart illustrates the differences in the perception of time of action and tone between the two hands (averaged together) in controls and (separated) in patients with CBS. Mean action (red bar) and tone (grey) binding values are displayed proportionally to their perceptual shift (error bars indicate mean standard error). Dashed lines indicate the veridical time of action and tone events. Significance level in pair-wise comparisons is indicated by ***P < 0.001 and **P < 0.01.

In healthy controls, perception of action and tone did not differ between hands [action: *t*(15) = −1.2, *P = *0.25; tone: *t*(15) = 0.19, not significant). Mean perception of action for the two hands was delayed relative to baseline by 23 ms, whereas perception of consequent tones was advanced relative to baseline by 56 ms (Fig. 2). These results are similar to binding measures observed previously in healthy young adults (Haggard *et al.*, 2002). In contrast, CBS markedly delayed the perception of action in the more-affected hand by 153 ms, but only 40 ms in the less-affected hand; tone perception advanced by 35 ms and 64 ms, respectively (Fig. 2).

Control and patient binding values were submitted to mixed-effects ANOVA, with Group (patients versus controls) as between-subject factor, and Event (action versus tone) and Hand (first hand tested, more-affected in patients versus other hand) as within-subject factor. Group × Hand [*F*(1,24) = 10.87, *P < *0.01] and Group × Event [*F*(1,24) = 4.67, *P < *0.05] interactions emerged. The critical result was a Group × Event × Hand interaction [*F*(1,24) = 4.4, *P < *0.05]. *Post hoc* two-tailed comparisons of patient data confirmed that action binding in the more-affected hand was greater than the less-affected hand [*t*(9) = 4.2, *P = *0.002], whereas tone binding did not differ between hands [*t*(9) = 0.85, not significant]. Across groups, action binding in the more-affected hand in patients was increased compared to action binding in controls [*t*(24) = 7.63, *P < *0.001], whereas in the less-affected hand it did not differ from controls [*t*(24) = 0.90, not significant]. These results indicate that action binding was specifically increased in the patients’ hand with greater volitional impairment.

Although action binding measures were enhanced in the more-affected hand in patients, this increase might interact with age or cognitive impairment, such as dementia or visuospatial deficits. In a subsidiary analysis, control and patient action binding data were entered into a mixed-design analysis of covariance, with age and Mini-Mental State Examination (Folstein *et al.*, 1975) as covariates. This additional analysis showed no interaction between hand and age [*F*(1,21) = 1.39, *P = *0.25] or between hand and Mini-Mental State Examination [*F*(1,21) = 2.58, *P = *0.12], but importantly a Group × Hand interaction remained significant [*F*(1,21) = 4.39, *P < *0.05].

### Increased binding of action related to clinical measures of abnormal voluntary control in corticobasal syndrome

We next tested whether the abnormally high action binding in the more-affected hand in patients with CBS was related to clinical motor symptoms. Alien limb, apraxia and asymmetric parkinsonism are prominent motor features among the clinical diagnostic criteria of CBS, and were common in our patients (Table 1).

To examine the relation between parkinsonian motor features and the enhanced action binding, we explored the correlation between abnormal binding and the Unified Parkinson’s Disease Rating Scale motor subscale III (Fahn and Elton, 1987) in the more-affected hand. Action binding and Unified Parkinson’s Disease Rating Scale did not correlate (Spearman’s rho = 0.19, not significant), in agreement with a previous study showing no alteration of binding in Parkinson’s disease (Moore *et al.*, 2010*a*).

Action binding in the more-affected hand positively correlated with-the-number of alien limb symptoms reported in that hand (Spearman’s rho = 0.79, *P = *0.007; Supplementary Fig. 1A), indicating that increased action binding in patients was related to severity of alien limb symptoms. Moreover, action binding in that hand negatively correlated with apraxia scores (number of gestures successfully performed; Spearman’s rho = −0.83, *P = *0.006; Supplementary Fig. 1B), indicating that increased action binding was also related to increasing severity of apraxia.

Although no association was found between severity of alien limb and apraxia scores (Spearman’s rho = −0.47, *P = *0.2), the results suggest that the increased binding of action captured a deficit shared by these motor abnormalities. We next link this quantitative measure of deficit with its underlying neural network, and in the ‘Discussion’ section we suggest a mechanism for this deficit.

### Grey matter changes associated with altered intentional binding

The analysis of grey matter was performed with voxel-based morphometry (Ashburner and Friston, 2000), implementing a general linear model with group (patients versus control subjects) as a categorical factor and action binding as a regressor of interest, adjusting for age and total intracranial volume. Group differences were observed at the lenient threshold of *P < *0.001 uncorrected (Supplementary Table 2), in line with previous voxel-based morphometry studies of CBS (Josephs *et al.*, 2008).

**Figure 3:**
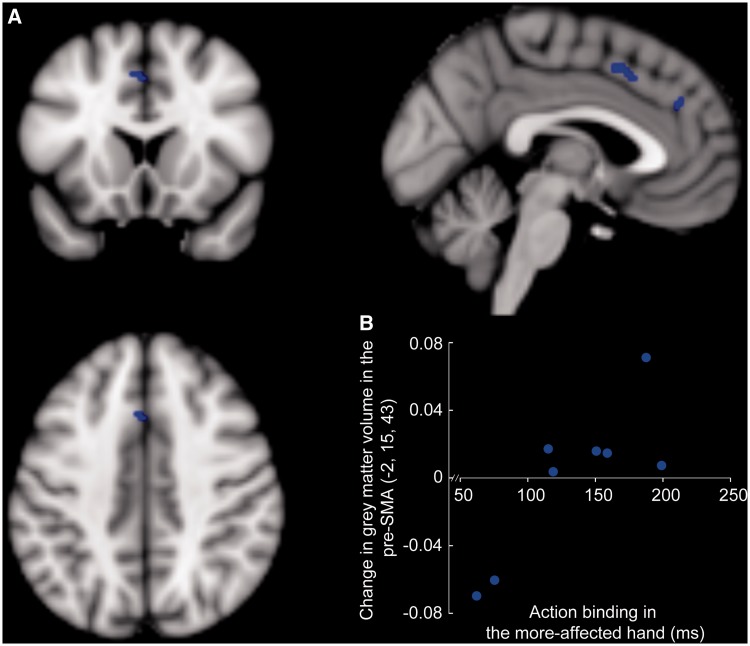
Grey matter correlates of action binding variability in patients with CBS. (**A**) Grey matter volume in the pre-SMA (*P < *0.05, FWE small volume corrected) and medial prefrontal cortex (*P = *0.05, FWE small volume corrected) correlated positively with action binding in patients (blue); overlaid on MNI 152 average brain (grey-scale). For illustration, significant voxels shown are at *P < *0.001, uncorrected. (**B**) Change in grey matter volume plotted against action binding in the more-affected hand in patients for the peak voxel in the pre-SMA (adjusted for group differences in action binding, total intracranial volume and age).

Our main hypothesis focused on the relation between awareness of action in patients, as quantified by action binding, and changes in grey matter. To this end, we specified an *a priori* region of interest encompassing the medial frontal and medial prefrontal areas (Amodio and Frith, 2006; Passingham *et al.*, 2010). Figure 3 illustrates the significant positive correlation between action binding and grey matter volume in the pre-SMA (*x = *−2, *y = *15, *z = *43; *P = *0.038, corrected) and a trend in the more anterior medial prefrontal cortex (*x = *3, *y = *38, *z = *28; *P = *0.055, corrected).

### White matter changes associated with altered intentional binding

We used tract-based spatial statistics (Smith *et al.*, 2006) of diffusion-weighted images to explore white matter changes associated with abnormal awareness of action. We examined mean diffusivity and fractional anisotropy, two alternative measures of white matter structure that are commonly used for assessing integrity of tracts. As before, for both mean diffusivity and fractional anisotropy, a general linear model analysis included Group (patients versus control subjects) as a factor and Action binding measure as a regressor of interest, adjusting for age. No significant group differences emerged for both measures. However, our main focus was on relating patients’ variability in action binding to changes in white matter microstructure.

**Figure 4:**
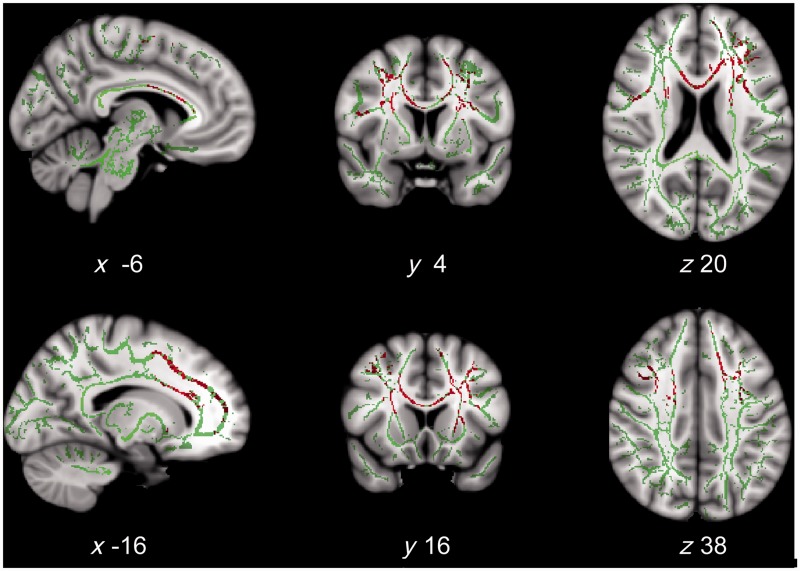
White matter correlates of action binding variability in patients with CBS. White matter tracts in which mean diffusivity positively correlated with action binding in the more-affected hand in patients (red; *P < *0.05, FWE corrected); overlaid on the mean fractional anisotropy skeleton (opaque green) and MNI 152 average brain (grey-scale). Slice coordinate is indicated. These tracts were adjacent to the medial frontal and medial and lateral prefrontal areas, and the anterior corpus callosum (tracts listed in Supplementary Table 3).

A positive correlation between action binding and mean diffusivity in patients was found in several white matter tracts (*P < *0.05, FWE corrected; tract listed in Supplementary Table 3). These included white matter adjacent to the pre-SMA and prefrontal cortex, the superior longitudinal fasciculus, and the anterior corpus callosum (Fig. 4). The correlations were positive, indicating increasing white matter deficit with increased (abnormal) binding. No correlation was observed with fractional anisotropy at the FWE corrected threshold *P < *0.05, but trends toward negative correlations between fractional anisotropy and action binding were observed at *P < *0.1, FWE corrected, in the anterior corpus callosum and prefrontal white matter tracts similar to those identified from mean diffusivity (Supplementary Fig. 2).

### Functional connectivity changes with altered intentional binding

Having established a focal grey matter structural locus in pre-SMA associated with altered binding of action, and white matter tract abnormalities convergent on this area and its connections, we examined the changes in functional connectivity of this area. We adopted a seed-based approach of resting-state functional MRI (Greicius *et al.*, 2003), using a modified dual regression analysis (Filippini *et al.*, 2009; see ‘Materials and methods’).

**Figure 5:**
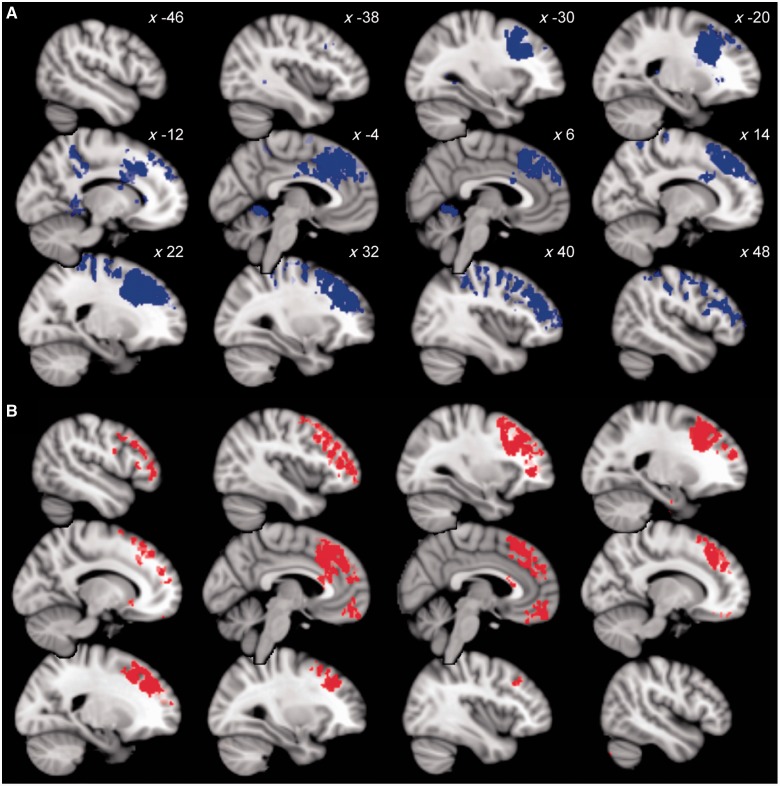
Functional connectivity of the pre-SMA associated with abnormal action binding. (**A**) Areas showing increased functional connectivity with the pre-SMA at rest in patients, relative to control subjects (blue; *P < *0.05, FWE corrected). Slice *x*-coordinate is indicated. A large fronto-parietal network showed increased coactivation with the pre-SMA, including the cerebellum, intraparietal sulcus, dorsal anterior cingulate cortex and lateral prefrontal cortex. (**B**) Voxels showing positive correlation between coactivation with the pre-SMA and action binding measures in patients (red; *P < *0.05, FWE corrected). Slices as in **A**. These correlations indicate a predominantly frontal cortical network associated with agency and the disorders of voluntary action, including alien limb phenomena and apraxia.

We first identified regions where the connectivity with the pre-SMA and medial prefrontal cortex significantly differed between groups. Relative to controls, patients with CBS showed wide areas of increased functional connectivity in a broad network of regions implicated in generating voluntary actions (Fig. 5A). These areas included bilateral dorsolateral prefrontal cortex, intraparietal sulcus, cerebellum and dorsal anterior cingulate cortex.

Functional connectivity between the pre-SMA and medial and lateral prefrontal cortex, including the dorsolateral prefrontal cortex, correlated with action binding (*P < *0.05, FWE corrected; Fig. 5B). This result indicates a central role of the pre-SMA within a distributed frontal-prefrontal network for voluntary action.

## Discussion

Our study combined an objective measure of the awareness of volitional actions that probes processes of motor control with multimodal neuroimaging techniques, to examine the mechanisms of impairments of voluntary action in CBS. We found abnormal binding in patients with CBS relative to control subjects, with increased temporal attraction of the perception of action toward a subsequent tone. The increase was specific to the more-affected hand and correlated with severity of alien limb and apraxia in that hand. Differences in binding were related to structural changes in pre-SMA grey matter, and the white matter underlying the pre-SMA and its connections to prefrontal cortex. The functional correlates of the behavioural changes were seen at rest, through changes in connectivity between the pre-SMA and the prefrontal cortex.

### Increased action binding in corticobasal syndrome

Intentional binding describes the perceived temporal attraction between a voluntary action and its sensory effect. Here, we found increased binding of action in patients with CBS with more severe volitional disorder. Although binding could capture the experience of individual actions and their effects (Walsh and Haggard, 2013), it is measured as an average across trials. Averaging across trials improves the precision, which is advantageous given the relatively low trial-by-trial resolution (each clock ‘minute’ position corresponding to an interval of ∼43 ms). The average increase in binding of action in patients with CBS thus reflected a consistent shift in the awareness of time of action, arising from changes in the experience of individual movements. Importantly, the magnitude of the effect (∼150 ms) was much larger even than the trial-by-trial resolution of the clock.

The behavioural change of binding as a result of CBS might reflect poor attention to the task or general motor abnormalities in patients. However, a critical internal control came from the results of the less-affected hand in patients, which were not different from control subjects. Moreover, increased action binding was linked to the severity of alien limb and apraxia, but not other motor features or cognitive impairments. Increased binding of action is therefore more likely to reflect the deficits in the awareness of action in the more-affected hand in CBS. Further, these results suggest that the sense of volition is specifically impaired in the clinically more-affected hand in patients with CBS, rather than a global impairment in agency.

The intentional binding paradigm has been used in healthy individuals and patient populations to measure the ‘sense of agency’, or -the- subjective experience that one controls one’s own actions and their consequences. Enhanced binding has been interpreted as an indicator of increased sense of control or agency (Moore *et al.*, 2010*a*). In contrast, here increased binding of action seemed to reflect increased deficits in sense of agency in patients with CBS. This apparent discrepancy is resolved by closer examination of the origins of binding, within the motor control theory, to which we turn next.

### Increased binding of action reflecting reduced precision of action signals

A key question arising from our behavioural data is why action binding was specifically enhanced with increasing severity of clinical volitional deficits in patients with CBS, with no change in tone binding. Motor control theory suggests that an efference copy of the motor command is used to predict the sensory consequence of one’s own action. Delays in this prediction process might lead to a late perception of action. However, as intentional binding is a relative measure, these delays would need to be specific to the case where the action causes a tone in the operant conditions, and not affect the baseline action. Such selectivity of perceptual delay is unlikely.

Action binding results from a cue integration of the action and sensory effect, whereby the action and its effect provide two separate cues for estimating the time of action (Wolpe *et al.*, 2013). The final estimate is then a weighted average of the action and tone cues, where the weight given to each cue depends on its reliability. On this basis, unreliable information about the action event would lead to an over-reliance on the tone cue when it is provided in the operant condition, and therefore increased binding of action. In contrast, cue integration is unlikely to support tone binding (Wolpe *et al.*, 2013), where unreliable cues are compensated by another mechanism (Waszak *et al.*, 2012), which might be spared in CBS.

Unreliable information about the time of action within the cue integration framework predicts that as the precision (i.e. reliability or the inverse of variability) of the action cue decreases, the weighting of the tone cue and consequently action binding would increase. That is, low precision of the action cue, reflected in increased variability of the estimates of action event alone in the baseline condition, would result in a proportional increase in binding of action. An additional analysis of our data confirms this prediction: the variability of time estimates in the baseline action condition and the extent of action binding in the more-affected hand were correlated across patients (Spearman’s rho = 0.65, *P < *0.05). This positive relation suggests that as the precision of information about the action decreases, action binding increases. Low precision in action signals could therefore account for increased binding of action in CBS.

Information about the time of one’s own voluntary action is provided by sensory signals of feedback from the moving body part and predicted sensation, and by internal volitional signals. Many of our patients had clinically detectable cortical sensory deficits (e.g. astereognosis or dysgraphesthesia), despite preservation of basic sensory modalities (light touch, pin prick, temperature and vibration). Moreover, increased noise in the process that predicts the sensory consequence of a voluntary action could lead to sensory deficits and low precision in the estimates of the time of one’s own action. However, such abnormal prediction process is also likely to have an impact on tone binding (Waszak *et al.*, 2012; Wolpe *et al.*, 2013), which we did not observe. Importantly, increased binding of action was not restricted to patients with sensory deficits. Together, the sensory deficits seem unlikely to be a sufficient cause of the low precision in the perception of time of action and the resultant increased action binding.

Normal perception of action is suggested to rely on volitional signals that drive voluntary behaviour according to the current goal. These signals could be built up during preparation for action, for example as measured by the ‘readiness potential’ (Lau *et al.*, 2007) that originates in the pre-SMA (Cunnington *et al.*, 2003). Our data suggest that in CBS, abnormalities in the neural processing within the pre-SMA or in its white matter connections, lead to unreliable volitional signals and a specific loss of information about actions. We propose that this increased noise or low precision of action signals is a major contributory mechanism to the volitional deficits of alien limb and apraxia in CBS. In the following sections, we link this mechanism to the functional anatomy and network changes in CBS, and discuss how it might contribute to the dissociable clinical phenomena of alien limb and apraxia.

### Association of medial frontal grey matter and abnormalities in voluntary action

In association with higher action binding in CBS, increasing grey matter volume was observed in the pre-SMA and more anterior medial prefrontal cortex. This association is critical: it not only underpins the seed-based connectivity analysis we used to identify a functional network for volition, but also confirms the link between binding, volitional deficits and the pre-SMA. Previous evidence for such a link was limited to temporary perturbations by transcranial magnetic stimulation in healthy volunteers (Moore *et al.*, 2010*b*). Interestingly, temporary lesions to the pre-SMA resulted in reduced tone binding with no effect on action binding. It is unclear, however, how temporary lesions induced by transcranial magnetic stimulation compare physiologically to neurodegenerative lesions.

The association of abnormal binding with more, rather than less grey matter volume, contrasts with a naive interpretation of neurodegeneration as simple tissue loss. However, focal increases in grey matter volume are observed in neurodegenerative diseases (Binkofski *et al.*, 2007; Reetz *et al.*, 2009). In the context of CBS, volume change may result from neurodegenerative pathologies or associated neuroplasticity that accompanies lesions (Rebeiz *et al.*, 1968; Gibb *et al.*, 1989). To understand functional networks, the volume or density of surviving neurons is not sufficient, and one should also consider the changes in connectivity with other brain regions.

### A disconnection syndrome underlying abnormalities in voluntary action

The mean diffusivity of white matter correlated with abnormal action binding in several frontal tracts. These included the tracts underlying both pre-SMA and lateral prefrontal areas, superior longitudinal fasciculus (providing frontoparietal connectivity) and anterior corpus callosum. Case studies have associated lesions in the anterior corpus callosum with volitional disorders of alien limb and apraxia in the non-dominant hand (Feinberg *et al.*, 1992; Scepkowski and Cronin-Golomb, 2003; Wheaton and Hallett, 2007). This large fibre bundle connects the motor areas of the two hemispheres (Witelson, 1989). Damage to this tract could thus lead to compromised transition of sensorimotor signals from the dominant to the non-dominant hemisphere.

Apraxia may represent a ‘disconnection syndrome’, whereby sensorimotor representations for voluntary movements are disconnected from the motor areas that execute them (Liepmann, 1905; Geschwind, 1965*a*, *b*; Wheaton and Hallett, 2007). The superior longitudinal fasciculus, white matter underlying motor areas, such as pre-SMA and the anterior corpus callosum, can carry sensorimotor representations for voluntary actions in frontoparietal motor areas, within and between the hemispheres. Our results of a group-level analysis of data from living humans provide a new layer of evidence for a disconnection syndrome underlying volitional deficits of alien limb and apraxia.

### Functional connectivity of the pre-supplementary motor area and prefrontal cortex in altered awareness and control of voluntary action

Functional connectivity of the pre-SMA at rest differed in patients with CBS with the medial prefrontal cortex, dorsolateral prefrontal cortex, dorsal anterior cingulate cortex and cerebellum. Many of these regions are the components of a robust ‘anterior salience’ network (Seeley *et al.*, 2007). Increased anterior salience connectivity has been reported in other neurodegenerative disorders, such as Alzheimer’s disease (Zhou *et al.*, 2010). The abnormally extensive functional connectivity might represent either a compensatory adaptation to disruption of a core network, or reduced efficiency within a broader frontoparietal network for control of voluntary action.

Connectivity between the pre-SMA and the medial and lateral prefrontal cortex was altered proportionally to abnormal binding. The interaction between the pre-SMA and dorsolateral prefrontal cortex is of special interest. The pre-SMA and dorsolateral prefrontal cortex have strong interconnections in comparative models (Luppino *et al.*, 1993), and both regions contribute to a network that supports voluntary behaviour, including the experience of intentions to act (Fried *et al.*, 1991; Lau *et al.*, 2004) and action decisions in the absence of external or learned cues (Deiber *et al.*, 1999; Rowe *et al.*, 2005).

How might impairments in this frontal network lead to both alien limb and apraxia? Although alien limb and apraxia co-occur in our patients and both relate to binding abnormality, they are dissociable clinical phenomena in CBS and other neurological disorders. Case studies of patients with focal lesions have shown that alien limb and apraxia have overlapping, but not identical, associations with underlying brain lesions (Scepkowski and Cronin-Golomb, 2003; Wheaton and Hallett, 2007). A disruption to their common neural substrate might therefore cause the manifestation of the two clinical phenomena. The association of these conditions with the medial frontal-prefrontal network for voluntary action, with its hub in the pre-SMA, suggests that this network is involved in both disorders. Supporting this, lesions in the pre-SMA can result in both alien limb and apraxia (Scepkowski and Cronin-Golomb, 2003; Wheaton and Hallett, 2007).

Studies of voluntary action have suggested that self- and externally-triggered actions are driven by distinct yet overlapping systems (Passingham *et al.*, 2010). We propose that the frontal network for awareness and control of voluntary action, centred on the pre-SMA, influences the balance between internal and external action systems, possibly through top-down control signals analogous to attentional control (Shallice, 1982). When an internally generated, non-habitual action is required for a certain goal, this frontal network could inhibit stimulus-driven and automatic behaviours, and drive the activation (or disinhibition) of self-generated motor plans through (pre)motor and striatal circuits.

The degree, localization and type of the disruption to this frontal network could shape its clinical manifestation. For example, a disconnection of this network from posterior parietal brain areas could lead to imprecise integration of spatio-temporal signals required for sequential, novel or intentional action schema. This type of disruption is more likely to result in apraxia in CBS (Borroni *et al.*, 2008). On the other hand, if the internally generated motor schemas are disproportionately imprecise, but habitual actions or affordances are relatively preserved, this would lead to an abnormal reliance on environmentally triggered motor schema for action selection. This type of disruption could lead to altered frontal activation (Schaefer *et al.* 2010) and a disinhibition of automatic movements (Blakemore *et al.*, 2002; Sumner *et al.*, 2007), resulting in the ‘exaggerated affordance’ effect for alien limb in CBS (McBride *et al.* 2013).

Resting state activity has itself been suggested to reflect conscious awareness and the sense of self (He and Raichle, 2009). Our study goes further in revealing a medial frontal-prefrontal network for awareness and control of voluntary action, with its disruption in CBS leading to low precision of action signals, and the volitional deficits of alien limb and apraxia. The importance of this network for awareness and control of voluntary action is underscored by the convergence of behavioural, anatomical and functional evidence.

## Limitations

There are several methodological and interpretative limitations to our study. The selection of the CBS patient cohort was limited to patients who could complete the intentional binding task and verify the event they judged after each block. This resulted in the exclusion of patients with marked dementia and pronounced visuospatial deficits, and patients in later stages of illness. Together with the prevalence of CBS, these inclusion criteria led to a relatively small sample size, and an increase in the risk of type II errors. In addition, most of the patients taking part in the study were on medication, and the effects of drugs on both behaviour and functional MRI are unclear. Moreover, as we do not yet have post-mortem diagnoses, we are unable to confirm the underlying pathology that caused CBS, although *in vivo* investigations indicate neurodegeneration, rather than vascular or metabolic disease.

The limitations of this study require a cautious interpretation of the data. However, they do not undermine the fundamental validity of our main results in elucidating the altered mechanisms for deficits in awareness and control of action in CBS. The finding of significant results in both the behavioural and imaging data was clear and specific. Furthermore, as drug treatment varied considerably across patients, medication is expected to add variability to the data, rather than being a systematic confound. Confirming neuropathological diagnoses (e.g. corticobasal degeneration or other neurodegenerative disease) is not critical to the interpretations, but we recognize that our results may not be simply generalized to the corticobasal degeneration population.

## Conclusion

We report a specific association between abnormal intentional binding and the volitional disorders of alien limb and apraxia in CBS, which we suggest result from low precision of internal volitional signals for actions. This behavioural abnormality correlates with focal structural changes in grey and white matter centred on the pre-SMA, and this region’s functional connectivity with the prefrontal cortex. The pre-SMA may therefore serve as a critical hub within a frontal network for awareness and control of voluntary action. Changes in the pre-SMA and its frontal connections can therefore affect both the objective capacity for voluntary control of action, and the subjective experience of agency. Understanding the functional anatomy and neurocognitive aetiology of volitional disorders in CBS may facilitate the development of new treatments for these highly disabling complications.

## Supplementary Material

Supplementary Data

## Abbreviations

CBS: corticobasal syndrome
SMA: supplementary motor area
