# Supplementary material for: The medial frontal-prefrontal network for altered awareness and control of action in corticobasal syndrome
Source: Brain. 2013 Nov 29;137(1):208–20. doi: 10.1093/brain/awt302 (PMC3891444; doi:10.1093/brain/awt302)
Supplement: Supplementary Data [file supp_137_1_208__index.html]

The medial frontal-prefrontal network for altered awareness and control of action in corticobasal syndrome — Supplementary Data 

# The medial frontal-prefrontal network for altered awareness and control of action in corticobasal syndrome

## Supplementary Data

files

**Files in this Data Supplement:**

- Supplementary Data - docx file
