# Supplementary material for: The medial frontal-prefrontal network for altered awareness and control of action in corticobasal syndrome
Source: Brain. 2013 Nov 29;137(1):208–20. doi: 10.1093/brain/awt302 (PMC3891444; doi:10.1093/brain/awt302)

**Supporting Information**

**Table S1.** Perceived times of key presses (action) and tones for all conditions across groups.

| **Group/**  **Hand** | **Condition** | **Event** | **Mean (SD) estimation error (ms)*** | **Mean (SD) binding: shift of operant estimation error from baseline (ms)** |
| --- | --- | --- | --- | --- |
| Controls, | Baseline | Action | -77 (52) |  |
| Right |  | Tone | - 7 (44) |  |
|  | Operant | Action | -48 (76) | 29 (42) |
|  |  | Tone | -65 (100) | -58 (95) |
| Controls, | Baseline | Action | -51 (36) |  |
| Left |  | Tone | -11 (48) |  |
|  | Operant | Action | -35 (60) | 16 (43) |
|  |  | Tone | -65 (72) | -54 (69) |
| Patients, | Baseline | Action | 4 (133) |  |
| more-affected |  | Tone | 37 (76) |  |
|  | Operant | Action | 157 (142) | 153 (51) |
|  |  | Tone | 2 (85) | -35 (105) |
| Patients, | Baseline | Action | 19 (95) |  |
| less-affected |  | Tone | 29 (60) |  |
|  | Operant | Action | 59 (108) | 40 (61) |
|  |  | Tone | -35 (95) | -64 (111) |

*Positive values indicate a delay in the perception of time of event estimated, and *vice versa* for negative values.

**Table S2.** Reduced gray matter volume in patients relative to controls (p<0.001, uncorrected). No differences were observed in the opposite contrast.

| **Area** | **Coordinates** | | |
| --- | --- | --- | --- |
|  | **x** | **y** | **z** |
| Primary somatosensory cortex | 56 | -7 | 34 |
| Dorsal-premotor cortex | 32 | 3 | 54 |
| Basal ganglia | 26 | 8 | -9 |
| Dorsolateral prefrontal cortex | -26 | 36 | 39 |

**Table S3.** White matter tracts with significant positive correlation between mean diffusivity and action binding in the more-affected hand in patients.

| **Tract** | **Threshold** | |
| --- | --- | --- |
|  | **p<0.01** | **p<0.05** |
| Corpus callosum | B | B |
| Forceps minor | B | B |
| Anterior corona radiata | B | B |
| Anterior thalamic radiation | L | B |
| Uncinate fasciculus | L | L |
| Inferior fronto-occipital fasciculus | L | L |
| Superior longitudinal fasciculus | B | B |
| Superior corona radiata | B | B |
| Internal capsule | - | B |
| External capsule | - | L |

B=bilateral; L=left hemisphere

**Figure S1.** Correlation between binding and clinical measures. (A) Alien Limb score plotted against action binding in the more-affected hand. These were significantly correlated across patients (Spearman’s rho=0.787, p=0.007). (B) As in A, but for action binding against apraxia scores, which also significantly correlated across patients (Spearman’s rho=-0.828, p=0.006).

A


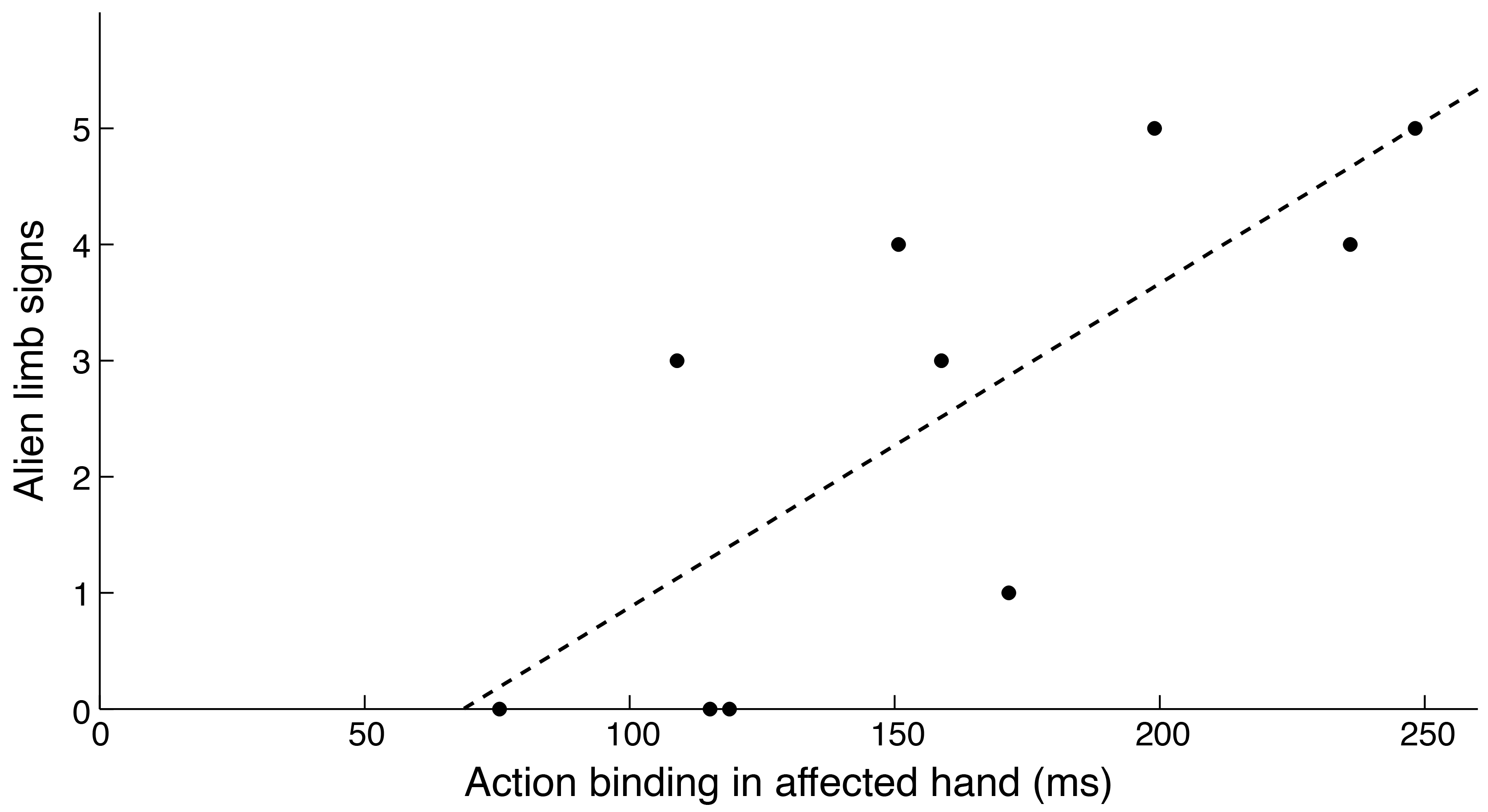


B


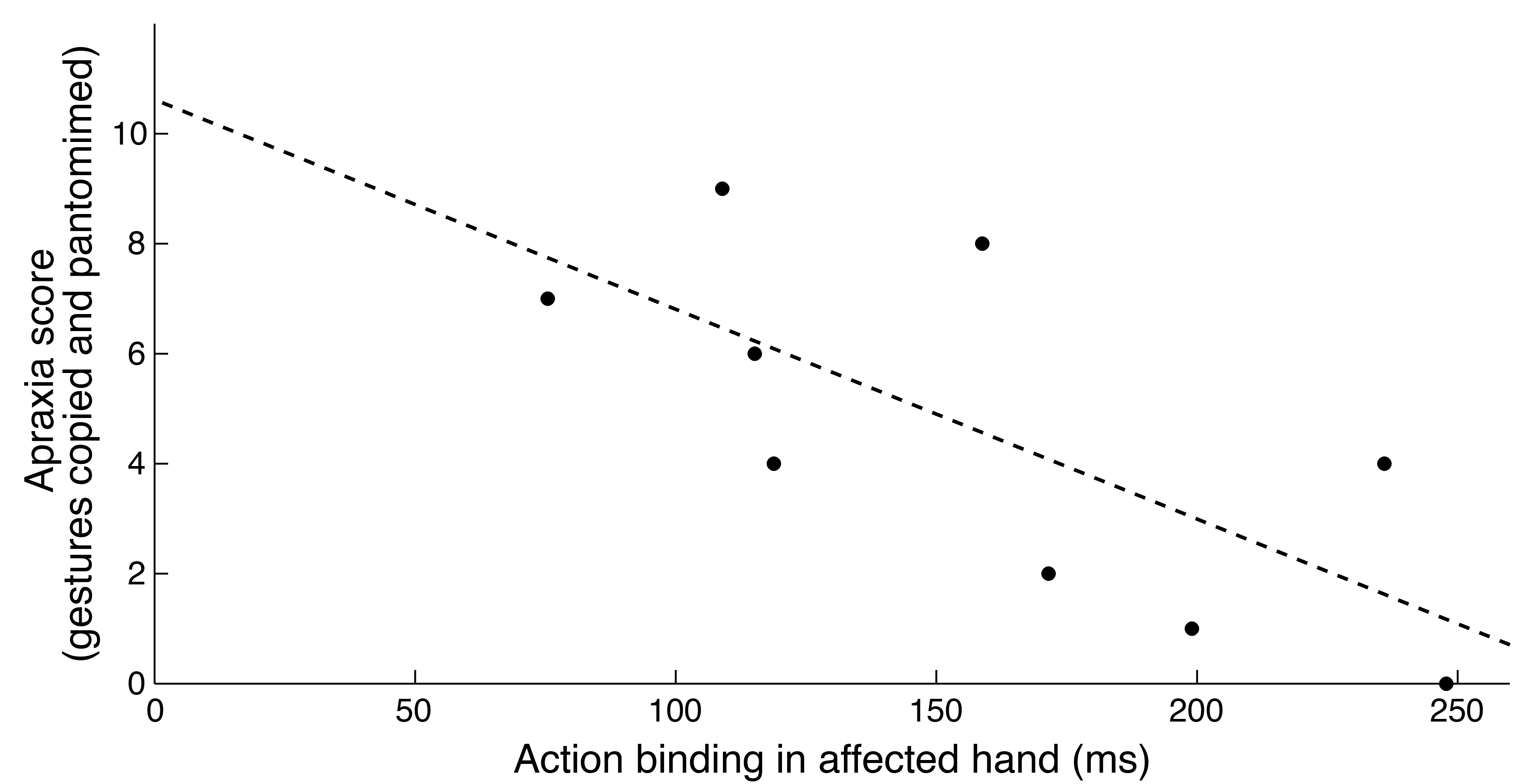


**Figure S2. Negative correlation between binding and Fractional anisotropy.** Analysis of trends at p<0.1 (FWE corrected) showing regions where patients’ action binding correlated negatively with fractional anisotropy (blue), overlaid on an MNI-152 average brain template (grayscale) and the mean fractional anisotropy skeleton (green). A negative correlation with fractional anisotropy indicates that the higher the binding score, the lower the fractional anisotropy, suggesting white matter abnormality in areas, mostly in the corpus callosum, similar to those identified in the positive binding-mean diffusivity correlation.


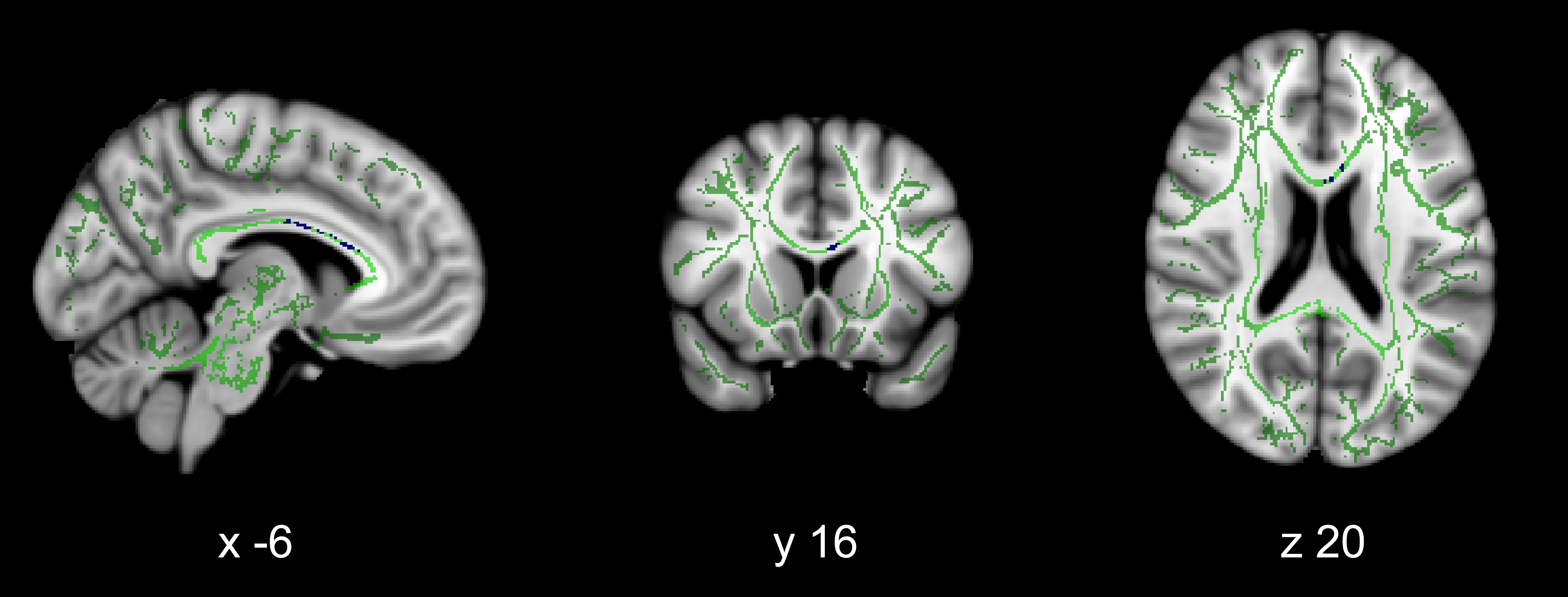

Supplement: Supplementary Data [file supp_awt302_brain-2013-00833-File008.docx]
